# A new method in thoracoscopic inferior mediastinal lymph node biopsy: a case report

**DOI:** 10.1186/1752-1947-3-96

**Published:** 2009-11-03

**Authors:** Maruf Sanlı, Ahmet F Isik, Bulent Tuncozgur, Levent Elbeyli

**Affiliations:** 1 Thoracic Surgery Department, Gaziantep University, Medical School, 27310 Şehitkami/Gaziantep, Turkey

## Abstract

**Introduction:**

We performed video-thoracoscopy with a video-mediastinoscope to conduct a mediastinal lymph node biopsy. Here, we discuss the various advantages of the method.

**Case presentation:**

A 56-year-old Turkish Caucasian man had been complaining of dyspnea on exertion, hacking cough, fever and continuous sweating for one and a half months. Thoracic computed tomography revealed enlarged paratracheal and aorticopulmonary lymph nodes, the largest of which was 1 cm in diameter and reticulo-micronodular interstitial infiltration extending symmetrically to the pleural surfaces in both pulmonary perihilar areas. Computed tomography supported positron emission tomography showed increased fluorodeoxyglucose retention in lymph nodes in both hilar areas (10R and 10L) (maximum standardized uptake values 5.6 and 5.7), and in the right lower paratracheal (4R) (maximum standardized uptake value 4.1) and right para-esophageal (8) (maximum standardized uptake value 8.9) lymph nodes. Pathological examination of the right lymph node number 8 biopsy using the video-mediastinoscope revealed the presence of granulomatous inflammation. No problems were observed during the postoperative period.

**Conclusion:**

The use of the video-mediastinoscope for inferior lymph node biopsy in thoracoscopy is an easy, safe and practical method, especially in patients with pleural adhesions.

## Introduction

Diagnosis of benign and malignant diseases involving the lungs and mediastinum can sometimes be difficult. Mediastinal lymph node biopsy plays an important role in the diagnosis of these diseases [1]. The video-endoscopic procedures used in thoracic surgery have brought about certain changes in the approach to the diseases [2]. Video assisted thoracoscopic surgery (VATS) used in mediastinal lymph node biopsy provides a high diagnostic success rate with a low morbidity rate. We report a case involving video-thoracoscopy using a video-mediastinoscope to conduct a mediastinal lymph node biopsy, and discuss the various advantages of the method.

## Case presentation

**Figure 1:**
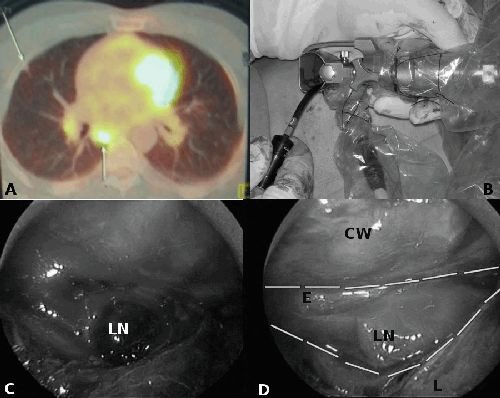
**Computed tomography supported positron emission tomography image that identified the fluorodeoxyglucose uptake in the para-esophageal lymph node (A)**. Image of the video-mediastinoscope used during the operation (B). Lymph node no. 8 can easily be seen by video-mediastinoscope after dissecting the pleural adhesions (C and D). LN, lymph node; E, esophagus; CW, chest wall; L, lung.

A 56-year-old Turkish Caucasian man had been complaining of dyspnea on exertion, hacking cough, fever and continuous sweating for one and a half months. He had been exposed to passive smoke for 25 years and biomass for 30 years. At the time of his admission, his body temperature was 36.6°C, pulse rate was 70/minute, arterial blood pressure was 110/70 mmHg and oxygen saturation was 93% (in room air). During auscultation of both pulmonary lower zones, end-expiratory rhonchi were audible. Erythrocyte sedimentation rate was 15 mm/hour and C-reactive protein was 9.67. Acid fast bacillus examination of a sputum specimen was found to be negative in three consecutive tests. Pulmonary function tests were normal. Thoracic computed tomography (CT) revealed enlarged paratracheal and aorticopulmonary lymph nodes with the largest node being 1 cm in diameter, and reticulo-micronodular interstitial infiltration symmetrically extending to the pleural surfaces in both pulmonary perihilar areas. CT supported positron emission tomography (PET) showed increased fluorodeoxyglucose (FDG) uptake in lymph nodes in both hilar areas (10R and 10L; maximum standardized uptake values (SUV max) 5.6 and 5.7), and in the right lower paratracheal (4R; SUV max 4.1) and right para-esophageal (8; SUV max 8.9) lymph nodes (Figure 1A). Fiber-optic bronchoscopy was normal. Transbronchial fine needle aspiration biopsy (FNAB) was performed on the 4R and 10R lymph nodes. Pathological examination of the specimens revealed chronic inflammation. A decision was made to sample the right lymph node number 8. Pathological examination of the biopsy from the right lymph node number 8 conducted by video-mediastinoscope revealed granulomatous inflammation. No problems were observed during the postoperative period. The chest tube was removed on the same evening as the operation when the drainage was minimal. The patient was discharged the following day.

General anesthesia was applied using a double lumen endotracheal tube, which ensured the collapse of the operated lung and the patient was placed in a 60° lateral position toward the front. The operative approach was performed through one thoraco-port. With the incision made in the 6th intercostal space on the posterior axillary line, the video-mediastinoscope (Richard Wolf, Germany) was advanced into the thoracic cavity (Figure 1B). Pleural adhesions were eliminated through blunt and sharp dissections and the lymph node in the paraesophageal region was reached. The video-mediastinoscope was further advanced into this area and the distal end was enlarged. Lymph node biopsy was performed using appropriate instruments (Figure 1C, D). The port was then used for inserting the chest tube.

## Discussion

After being widely accepted by the thoracic surgery community, VATS has been used as an important diagnostic and staging instrument in many clinics [3]. Its diagnostic value in mediastinal diseases is between 85% and 100%. VATS is often used for inferior mediastinal lymph node sampling [4]. FNAB results were obtained from lymph nodes 10R and 4R, which were found to have relatively lower retention rates in PET screening and so a diagnosis could not be established. For diagnostic purposes, a biopsy of the right paraesophageal lymph node with a higher retention rate was planned via the thoracoscopic method. Recent innovations in the optics, video systems and endoscopic instruments have given rise to an increase in thoracoscopic surgical procedures and allowed for an easier operation. With this aim, we conducted the thoracoscopy during which we used a video-mediastinoscope.

The 60° lateral position toward the front was aimed at shifting the lung in order to open the posterior mediastinal area. However, severe adhesions did not allow for this. The video-mediastinoscope that we used provided a clear advantage compared to the classic VATS instruments. In comparison to classic VATS, the long and wide channel maintained by the shaft of the video-mediastinoscope was advanced to the biopsy field to provide a safe route while preventing pulmonary parenchymal injuries during the entry and exit of the instrument. The ability to open the distal end allowed for moving the lung from the biopsy area and provided a wider area for the necessary manipulations. Because the proximal end of the blades in the entry is not widely separated while the distal end is widely separated, the probability of intercostal nerve injuries is much lower. When necessary, two different instruments were used in the wide channel of the video-mediastinoscope. Thus, existing adhesions were relieved more easily and rapidly than would be possible with a classic VATS. In addition to video imaging, the wide channel allowed direct observation. Video imaging also permitted recording and this was used as a training instrument just as in all VATS systems. Although Cerfolio et al. [5] used one port to access lymph nodes number 5 and 6, in the classic VATS, an average of 2-3 ports is required and additional personnel are needed during the operation. In our patient, we used different instruments through one port. At the end of the operation, the port was used for inserting the chest tube. We did not come across thoracoscopic inferior lymph node biopsy conducted via video-mediastinoscope in the literature.

Esophageal endoscopic ultrasound fine-needle aspiration (EUS-FNA) is a minimally invasive technique that allows sampling of subcarinal lymph nodes, paraesophageal lymph nodes and inferior pulmonary ligament lymph nodes for the staging and diagnosis of patients with lung cancer [6]. Use of EUS-FNA should decrease the necessity for thoracoscopic procedures, which need general anesthesia and a double lumen endotracheal tube.

## Conclusion

Using the video-mediastinoscope in thoracoscopy for inferior lymph node biopsy is an easy, safe and practical method, especially in patients with pleural adhesions.

## Abbreviations

ARB: acid resistant bacillus; CT: computed tomography; EUS-FNA: esophageal endoscopic ultrasound fine-needle aspiration; FDG: fluorodeoxyglucose; FNAB: fine needle aspiration biopsy; PET: positron emission tomography; SUV: standardized uptake value; VATS: video assisted thoracoscopic surgery.

## Consent

Written informed consent was obtained from the patient for publication of this case report and any accompanying images. A copy of the written consent is available for review by the Editor-in-Chief of this journal.

## Competing interests

The authors declare that they have no competing interests.

## Authors' contributions

MS was involved in the management of the patient as well as writing the case reports. AFI took the photographs. BT and LE were involved in the correction of the manuscript as well as general supervision. All authors read and approved the final manuscript.
